# Unusual Novel SnoRNA-Like RNAs in *Drosophila melanogaster*

**DOI:** 10.3390/ncrna1020139

**Published:** 2015-07-13

**Authors:** Alberto Agrisani, Hakim Tafer, Peter F. Stadler, Maria Furia

**Affiliations:** 1 Department of Biology, University of Naples “Federico II”, Complesso Universitario Monte Santangelo, via Cinthia, I-80126 Napoli, Italy; E-Mails: alberto.angrisani@unina.it (A.A.); mfuria@unina.it (M.F.); 2 Institut für Biotechnologie, Universität für Bodenkultur, Muthgasse 18, A-1190 Wien, Austria; E-Mail: htafer@gmail.com; 3 Bioinformatics Group, Department Computer Science; Interdisciplinary Center for Bioinformatics; German Centre for Integrative Biodiversity Research (iDiv), Halle-Jena-Leipzig; University Leipzig, Härtelstrasse 16-18, D-04107 Leipzig, Germany; 4 Max Planck Institute for Mathematics in the Sciences, Inselstraße 22, D-04103 Leipzig, Germany; 5 Fraunhofer Institute for Cell Therapy and Immunology, Perlickstrasse 1, D-04103 Leipzig, Germany; 6 Department of Theoretical Chemistry, University of Vienna, Währingerstrasse 17, A-1090 Vienna, Austria; 7 Center for RNA in Technology and Health, University of Copenhagen, Grønnegårdsvej 3, DK-1870 Frederiksberg C, Denmark; 8 Santa Fe Institute, 1399 Hyde Park Road, Santa Fe, NM 87501, USA

**Keywords:** snoRNA, long non-coding RNA, sno-lncRNAs, Drosophila

## Abstract

A computational screen for novel small nucleolar RNAs in *Drosophila melanogaster* uncovered 15 novel snoRNAs and snoRNA-like long non-coding RNAs. In contrast to earlier surverys, the novel sequences are mostly poorly conserved and originate from unusual genomic locations. The majority derive from precurors antisense to well-known protein-coding genes, and four of the candidates are produced from exon-coding regions. Only a minority of the new sequences appears to have canonical target sites in ribosomal or small nuclear RNAs. Taken together, these evolutionary young, poorly conserved, and genomically atypical sequences point at a class of snoRNA-like transcripts with predominantly regulatory functions in the fruit fly genome.

## 1. Introduction

Small nucleolar RNAs (snoRNAs) are among the few ancient non-coding RNA (ncRNA) classes that predate the radiation of Eukaryotes. Their biology has been well studied and often reviewed [1,2,3,4]. Their primary function is to act as “address labels” for small nucleolar ribonucleotide protein (snoRNP) complexes, defining the specific locations at which chemical modifications are introduced into their RNA targets by means of complementary base pairing. There are two main types of snoRNAs, distinguished by characteristic sequence boxes as well as characteristic secondary structures: Box C/D snoRNAs, with two short motifs C (RUGAUGA) and D (CUGA), and a short helix connecting the 3′- with the 5′-end, direct 2′-O-methylation of nucleotides. Box H/ACA have a more elaborate secondary structure, usually comprising two hairpin structures separated by motif H (ANANNA) and featuring the sequence ACA three positions from their 3′-end. H/ACA snoRNPs catalyze the specific conversion of uridines into pseudouridines. Both classes of snoRNAs primarily target ribosomal RNAs (rRNAs) and spliceosomal small nuclear RNAs (snRNAs), thereby influencing stability, folding, and interactions of their targets. An increasing number of non-canonical functions have been discovered in recent years. These include the regulation of mRNA editing, alternative splicing, and gene silencing through microRNA-like mechanisms. While some snoRNAs are “orphans” in the sense that they have no known targets, others seem perform both traditional and non-canonical functions, see [4,5,6] for recent reviews. Recent evidence has implicated snoRNAs as important regulators of cellular functions—dysfunction of snoRNAs in particular appears to have a key role in oncogenesis [7,8].

Almost all animal snoRNAs are encoded in introns. Nevertheless, the expression patterns of snoRNAs are highly variable, as has been observed particularly in cancer samples [9]. In a recent study [10], we analyzed in some detail the genomic organization of annotated snoRNAs in the fruit fly *Drosophila melanogaster* and found that genes involved in cell division and cytoskeleton organization are preferred snoRNA hosts. SnoRNAs exhibit dynamic developmental expression patterns that are often distinct from the temporal profiles of other snoRNAs encoded in introns of the same host gene. This decoupling of expression levels may be achieved by alternative splicing and alternative processing of different isoforms [11]. The many potential regulatory roles of snoRNAs also suggest that—as in the case of microRNAs—novel snoRNA families may arise *de novo* and take on lineage-specific roles.

The snoRNA complement of fruit flies has been studied extensively in the past [12,13]. Earlier studies, in particular, used computationally predicted candidates derived from known targets [14,15,16,17]. Many of the fruit fly snoRNAs appear in clusters, more precisely in different introns of a single host gene [10,18], albeit in sometimes unusual arrangements. Several snoRNAs, for example, are encoded in introns in alternative splicing and polyadenylation variants of the pseudouridine synthase gene [19]. In some cases, however, the transcript structure deviates drastically from the vertebrate standard. *MeU1b-A234*, for example, derives from a longer, unspliced precursor transcribed from the reverse strand of the *egr* gene, a key regulator of cell differentiation, apoptosis and immune response in flies [20].

Motivated by such aberrant examples, we report here on a computation-based search for snoRNAs and snoRNA-like RNAs in the *D. melanogaster* genome, which includes snoRNA candidates in unusual genomic contexts as well as candidates that exhibit only a very limited phylogenetic distribution. Such snoRNAs are of particular interest because they plausibly constitute lineage-specific regulators rather than canonical guide RNAs.

## 2. Materials and Methods

### 2.1. Computational Analysis

We combined bioinformatic approaches based on different screening parameters to consecutively scan the fruit fly genomic sequence, with the aim to enhance the sensitivity and specificity of our survey.

We used SnoScan-0.99b with default parameters [21] to scan the complete *D. melanogaster* genome. This tool recognizes the terminal stem, the characteristic box C and D sequence motives, and the sequence complementary to putative target sites. It is therefore limited to box C/D snoRNAs with known target sites. SnoRNAs with a SnoScan-0.99b higher than 15 were kept. To alleviate this limitation, we used every position in the *D. melanogaster* rRNAs as a putative methylation site. Genomic regions surrounding these initial candidates were re-evaluated with snoReport-1.0 [22] using default parameters, to increase the sensitivity of the search. This program is designed to improve the prediction of snoRNAs with non-canonical antisense elements, and thus with no obvious RNA target, and can thus also recognize orphans of either C/D and H/ACA family. This feature is particularly relevant since, due to frequent degeneration of consensus boxes observed for *Drosophila* snoRNAs, experimentally validated candidates are often not included in the list of those displaying top scores with the SnoScan-0.99b program [16]. Considering the frequent clustering of snoRNAs, screening with snoReport also allows the discovery of H/ACA snoRNAs flanking C/D putative genes, a type of organization not common in *Drosophila*.

Candidate sequences were investigated for homologs in other drosophilid genomes using the available genome-wide multiple sequence alignments and a blast search. Consensus secondary structures of snoRNA candidates were analyzed with RNAalifold program [23] to check for conserved typical secondary structure.

We used RNAsnoop [24] and PLEXY [25] to predict possible targets for the novel candidate sequences. Both programs were run with default parameters.

A diagram of the work flow is represented in Figure S3.

### 2.2. RNA Extraction and Analysis

Canton S was used as wild-type strain in all experiments. Total RNA from 0–24 h mixed-stage embryos, a mixed population of first-second-third instar larvae, mixed-stage pupae and adults of both sexes at 4 days after eclosion, was extracted using TRI Reagent (Sigma-Aldrich, St. Louis, MO, USA) following the manufacturer’s instructions.

RNA was treated with TurboDNase (Life Technologies, Carlsbad, CA, USA) and phenol:chloroform extraction, 1 μg RNA was reverse transcribed using SuperSript III RT (Life Technologies) using the manufacturer’s recommended conditions and diluted 1:10. To check for gDNA elimination, 1:10 dilutions of RT plus and minus reactions were used as a template for amplification of 7SL-RNA by qualitative PCR using DreamTaq (Life Technologies) and applying the manufacturer’s recommended conditions; negative amplification of RT minus reaction was used to verify the complete digestion of gDNA.

For Northern blot analysis, 6 μg of total RNA was electrophoresed and transferred onto Hybond-NX (GE Healtcare, Fairfield, CT, USA) membranes. Probes were produced by amplifying PCR gDNA fragments (0.3–0.5 kb in length) spanning the detected sequences. PCR fragments were then 32P-labeled using the Nick Translation Kit (Hoffmann-La Roche, Basel, Switzerland). DNA extraction, manipulation and labelling, RNA electrophoresis, and blotting were carried out according to [26]. The size of RNAs was determined by using high range and low range RNA molecular weight markers (Life Technologies)

Quantitative real-time RT-PCR (qRT-PCR) experiments were performed in triplicate using iQ5 Multicolor Real-Time PCR Detection System (Bio-Rad, Hercules, CA, USA) as previously described [20]. All PCR reactions were carried out in a final volume of 15 μL using 1 μL of diluted cDNA, 7.5 μL of 2X SYBR-Green (Bio-Rad) and 5 pmol of each primer. All primer sequences were designed using Primer 3 software (bioinfo.ut.ee/primer3-0.4.0/) [27] and are available under request. *RpL32* was used as endogenous control for sample normalization. qRT-PCR experiments were restricted to snoRNAs in the canonical size range that do not overlap exons.

Quantitative PCR analysis was performed using the $2^{-\Delta \Delta CT}$-method [28]. Sequencing was performed by the external firm PRIMM (Milano, Italy).

All newly identified sequences were named according to the FlyBase Nomenclature rules that are found at http://flybase.org/wiki/FlyBase:Nomenclature [33]. These rules were extended to scaRNAs.

## 3. Results

**Figure 1:**
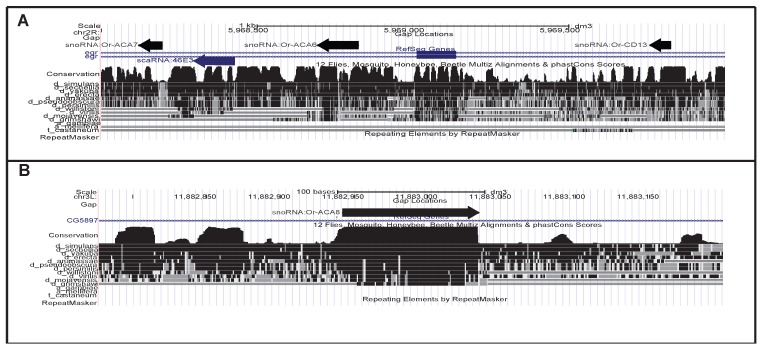
Two examples of novel snoRNAs. (**A**) Two box H/ACA and one box C/D snoRNAs were detected antisense to the *eiger* (*egr*) gene. Presumably, they derive from a common precursor with the scaRNA *MeU1b-A234* (*scaRNA:46E3*); (**B**) *Or-ACA8* is the best conserved among the novel snoRNAs, dating back at least to the ancestor of the *Drosophila* genus.

We employed different computational approaches to extend the current snoRNA annotation of drosophilids. We identified and experimentally validated (by Northern blot and RT-PCR) fifteen novel genes, whose main features are summarized in Table 1. All identified sequences were named following the FlyBase nomenclature rules. Sequences of all identified specimens were well conserved within the melanogaster subgroup, but none of them has a recognizable homolog outside the *Drosophila* genus, see Figure 1 and Figure S1. Only snoRNA *Or-ACA8* and possibly *Or-CD15* appears to be present in the entire *Drosophila* genus, while the other genes are later innovations (For clarity, we omit the prefixes *snoRNA:* and *scaRNA:* from gene names throughout the text.). Even more surprisingly, only a minority of the sequences are under stabilizing selection, as measured by phastcons [29] scores displayed in the UCSC genome browser. Among these, only *Or-ACA8* and, to a lesser extent, *Or-ACA6*, *Or-CD13*, *Or-CD14*, *Or-CD15*, and *Me28S-C2789*, show appreciable levels of sequence conservation, even though the conserved parts do not cover the entire length of the ncRNAs.

Ten of the transcripts conform to the typical length range of snoRNAs, while five are much longer and may constitute snoRNA-like long non-coding RNAs. Such sno-lncRNAs that feature snoRNA-like ends have been found as predominantly species-specific transcripts in mammals [30,31], making their presence in flies a credible proposition. The sequences of 11 well-characterized transcripts were deposited in GenBank. For the remaining four snoRNA-like lncRNAs the true extent of the RNAs remains unknown. These sequences can be found in the supplementary material together with Northern blots.

**Table 1:** Summary of newly identified snoRNAs. Subscripts to the host gene identify the intron, superscripts indicate an exonic position, and * indicates that the snoRNA overlaps a splice junction.

Name	Class	Length	Genomic Location			Accession	Host Gene	Opposite to
*snoRNA:Or-ACA6*	ACA	137	chr2R	5,968,693–5,968,830	-	KJ808674	*Uhg46E3*	*egr*_1_
*snoRNA:Or-ACA7*	ACA	81	chr2R	5,968,118–5,968,199	-	KJ808675	*Uhg46E3*	*egr*_1_
*snoRNA:Or-CD13*	CD	69	chr2R	5,969,758–5,969,827	-	KJ808676	*Uhg46E3*	*egr*_2_
*snoRNA:Or-CD15*	CD	96	chr3L	1,667,151–1,667,247	-	KJ808681	*RpL23A*^3*^ 4	
*snoRNA:Me28S-A2629*	CD	140	chr3L	10,530,718–10,530,858	+	KJ808678	*A2bp1*^2^	
*snoRNA:Or-ACA8*	ACA	93	chr3L	11,882,942–11,883,035	+	KJ808677		*CG5897*_1_
*snoRNA:Or-CD14*	CD	126	chr2L	2,014,449–2,014,575	-	KJ808682		*CG4238*_1_
*snoRNA:Me28S-C2789*	CD	148	chr2L	21,034,775–21,034,923	+	KJ808683		*CG42238*_1_
*snoRNA:Me18S-G1506*	CD	147	chr2L	16,561,050–16,561,197	-	KJ808679		*CG42389*_1_
*scaRNA:MeU1:95C-A24*	CD	106	chr2L	901,976–902,082	-	KJ808680		
*snoRNA:Or-ACA9*	ACA	600	chr3R	11,297,723–11,298,370 †‡	-	KJ808684	*AdamTS-A*_1_	
*snoRNA:Or-CD16*	CD	400	chr2L	3,504,032–3,504,182 ‡	-	Suppl. data	*tim*^2^	
*scaRNA:MeU4:25F-C137*	CD	600	chr3R	1,410,224–1,410,366 ‡	-	Suppl. data	*CG2926*^3^	
*snoRNA:Me28S-C993*	CD	400,1200	chr3L	10,189,262–10,189,354 ‡	-	Suppl. data	${\mathrm{ect}}_{2}^{*}$	
*snoRNA:Or-CD17*	CD	500	chr2R	12,958,091–12,958,193 ‡	+	Suppl. data		${\mathrm{CG30460}}_{3}^{*}$

† The start of the transcript is only approximate, due to a discrepancy between the deposited and verified (KJ808686) genomic sequences. ‡ The snoRNA coordinates were determined with *in silico* methods, while the transcript length is deduced from Northern Blots.

The majority of newly identified snoRNAs had no obvious target on rRNAs or snRNAs and were thus classified as orphans. Plausible target sites in rRNAs are predicted only for the three methylation guides *Me28S-A2629*, *Me28S-C2789*, *Me18S-G1506* as well as for the long transcript *Me28S-C993*. The two methylation guides *MeU1:95C-A24* and *MeU4:25F-C137* might target snRNAs and thus are tentatively classified as scaRNAs. In particular, *MeU1:95C-A24* is predicted to recognize all *D. melanogaster* U1 snRNA isoforms, see also Table S1.

Targets for the orphan snoRNAs were then sought genome-wide using RNAsnoop and PLEXY with default parameters. No plausible target site was found for any of the box H/ACA snoRNAs. This may be explained by their reduced sequence length, which impedes the formation of the stabilizing upper stem upon binding. We hypothesize, therefore, that these box H/ACA may have non-canonical functions, such as the production of small RNAs. In contrast, we identified putative targets for all five orphan box C/D snoRNAs. The best interactions are compiled in Table S2.

Surprisingly, except for two specimens (*Me28S-A2629* in *A2bp1* and the long *Or-ACA9* in *AdamTS-A*) none of the new transcripts is located in the expected intronic position. Instead, they exhibit peculiar genomic arrangements that may have precluded their detection in previous screens. Eight snoRNAs were found antisense to protein-coding genes.

A particularly interesting case are the snoRNAs *Or-ACA6*, *Or-ACA7* and *Or-CD13* that map close to each other in the vicinity of the previously identified *MeU1b-A234* (formerly known as *scaRNA:46E3*) [20]. The tight clustering of these snoRNAs is suggestive of the polycistronic transcription common to Uhgs. At least *MeU1b-A234* derives from a longer precursor antisense to *egr* [20]. We therefore name this new snoRNA cluster and its putative precursor *Uhg-46E3*. It is atypical in several respects. First, it is heterogeneously composed of an equal number of H/ACA and C/D specimens. All of its components show very little sequence conservation and are very different, so they cannot have originated through a series of local gene duplications. Third, the whole cluster overlaps, with opposite polarity, the *eiger* gene (*egr*), a member of the TNF family involved in programmed cell death and immune response acting through the JNK pathway [32]. Although unique among previously annotated Uhgs, this antisense arrangement is highly conserved across the melanogaster subgroup (see Figure S1), suggesting that it might have functional significance, possibly directly affecting *egr*. This hypothesis is supported by the earlier observation that *MeU1b-A234* is significantly up-regulated in mutants in which *egr* transcription was reduced [20].

Another four snoRNAs (*Or-CD15*, *Or-CD16*, *MeU4:25F-C137*, and *Me28S-C993*) are released from an exon of protein-coding genes transcribed with the same polarity. The existence of these putative snoRNAs has been validated by Northern blots (Figure S2). Similar exonic arrangements were previously reported for a few Drosophila snoRNAs (*snoRNA:660*, *snoRNA:83E4-5*, *snoRNA:Me28S-G2596*, *snoRNA:Or-CD11a*, *snoRNA:Or-CD11b*, *snoRNA:Or-ACA2*), see http://Flybase.org [33]. Three of them derive from coding regions, posing intriguing questions about their expression strategy and the possibility of an influence on host mRNA processing in a way similar to yeast U18 snoRNA [34].

**Figure 2:**
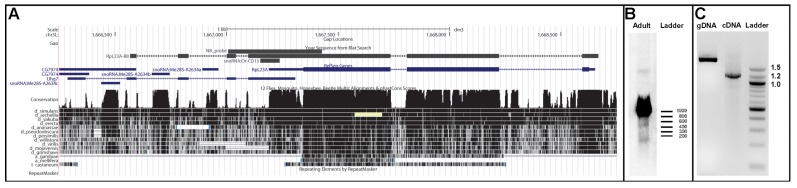
Genomic organization and expression strategy of *Or-CD15*. (**A**) Screen shot of the *RpL23A*/*Uhg7* region taken from the UCSC genome browser. The RpL23A-RA and Uhg7 transcripts are depicted in blue, the location of *Or-CD15* and the newly identified transcript RpL23A-RB are shown in black. Below, sequence conservation as measured by phastcons and the coverage of a multiple genome alignment is indicated; (**B**) Northern blot analysis of adult fruit fly RNA. The genomic position of the utilised probe is outlined in (**A**). The band at about 100 nt corresponds to snoRNA *Or-CD15*; sizes are indicated by an RNA molecular weight ladder (RiboRuler Low Range, Life Technologies); (**C**) PCR experiments using primers annealing to *RpL23A* exon 1 and *Uhg7* exon 4; amplification of the genomic DNA (gDNA) produces the expected fragment of about 2 kb; the same primers used in RT-PCR experiments successfully amplified a fragment of 1.2 kb, representative of the new *RpL23A-RB* transcript. In this transcript, *RpL23A* and *Uhg7* sequences are fused to each other. On the right, a DNA molecular weight ladder (100bp DNA ladder, New England BioLabs, Ipswich, MA, USA) is given.

The biogenesis of the snoRNA *Or-CD15* is closely related to other members of the *Uhg7* cluster. It originates from an exon shared by *RpL23A* and *Uhg7* (Uhg7-RA FlyBase ID: FBtr0300232) genes and appears to be particularly puzzling. Indeed, *Uhg7* itself exhibits a complex genomic arrangement, since sequence of a long annotated EST (GenBank: BP542227) indicates that it partially overlaps with both *RpL23A* and *CG7974* flanking genes, Figure 2. To better explore the expression strategy of snoRNA *Or-CD15* we performed RT-PCR experiments by using primers annealing on the *RpL23A* exon 1 and *Uhg7* exon 4, respectively. This approach lead to productive amplification of a cDNA fragment (Figure 2), whose sequencing defined a previously undescribed long transcript of the *RpL23A* locus (RpL23A-RB, accession number KJ808685) that ends at an alternative 3′-distal site and extends over *Uhg7* sequences. Structure of this cDNA thus revealed that *RpL23A* and *Uhg7* can be included together in a common, long, spliced RNA precursor. However, we found that this precursor still retained the unspliced *Or-CD15* sequence, suggesting that release of this snoRNA might depend on a rare alternative splicing event or, alternatively, by a splicing-independent mechanism [11,34,35].

An unusual arrangement was noticed also for *MeU1:95C-A24*, which maps, as an independent singlet, only about 300 bp upstream of the divergently transcribed snRNA *U1:21D* that represents one of its potential targets (Figure S1). This type of genomic organization raises the possibility that this gene pair might share the same upstream regulatory region and be coordinately regulated.

In the light of recent suggestions that C/D snoRNA abundance can oscillate according to circadian rhythm [36], it is also interesting to note that a new specimen, *Or-CD16*, originates from *timeless* (*tim*), a regulatory gene involved in the response to light stimulus.

Northern blot analysis often identified transcripts whose size was longer than that of canonical snoRNAs (see Figure S2), in line with the finding that regardless of their genomic arrangement, snoRNAs are often part of longer stable transcripts, possibly related to sno-lncRNAs [30,31].

**Figure 3:**
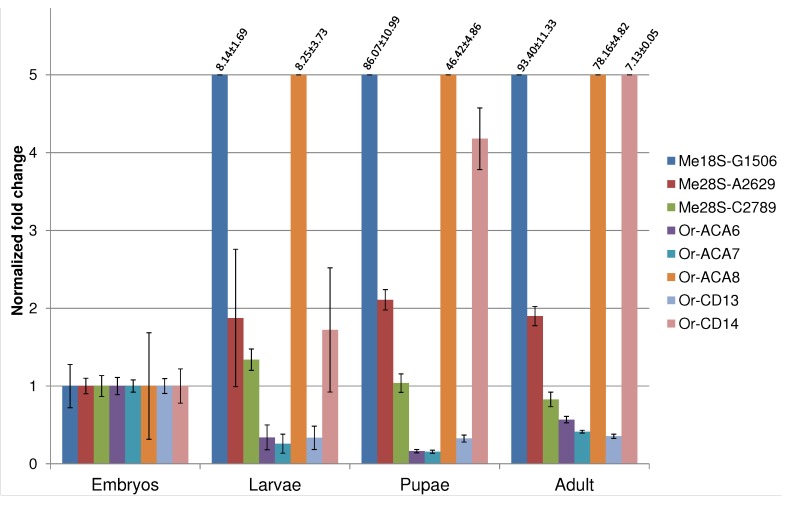
Developmental expression profiles of the eight novel snoRNAs that are within the typical size range for snoRNAs and that do not overlap known exons.

We evaluated the developmental expression profile of those eight newly identified snoRNAs by qRT-PCR that are within the expected size range for canonical snoRNAs and that do not overlap exons of other transcripts. To account for experimental errors, sex-related bias and stochastic fluctuation in expression profiles, we define a snoRNA as *developmentally regulated* if it shows a fold change of at least 2× relative to the embryo reference stage. According to this rule, and in agreement with previous data [10], only two of the assayed snoRNAs (*Me28S-A2629* and *Me28S-C2789*) show a time course of expression that is not dependent on the developmental stage. All snoRNAs hosted in *Uhg-46E3* (*Or-ACA6*, *Or-ACA7*, and *Or-CD13*) show a reduction of expression along the development. The remaining snoRNAs (*Me18S-G1506*, *Or-ACA8* and *Or-CD14*) are all constantly up-regulated along development, reaching maximum expression at adult stage (see Figure 3). These data confirm the fine modulation of snoRNA expression.

## 4. Discussion and Conclusions

We have conducted a computational survey for novel snoRNAs in *D. melanogaster* using a combination of established methods. In contrast to earlier work, we have focused on recent innovations and poorly conserved snoRNAs, *i.e.*, deep sequence conservation did not play a major role in our approach. We discovered 15 novel snoRNAs and snoRNA-like long non-coding RNAs. Surprisingly, the majority of these transcripts are located in a genomic context very atypical for snoRNAs, namely antisense to protein-coding genes or overlapping coding sequences in the sense direction. Three of the novel snoRNAs originated from the novel polycistronic untranslated host gene *Uhg-46E3*, antisense to the regulatory gene *eiger*. In another case, the expression of a non-coding host gene can be coupled to that of the upstream *RpL23A* gene. This arrangement is highly reminiscent of the gene structure previously observed for the *Drosophila* pseudouridinylase *mfl* and the associated *Uhg6* snoRNA host genes [19]. The complexity of these genomic loci highlights the complexity underlying snoRNA regulation and indicates that the expression strategy of some previously annotated Uhgs needs to be revised.

Taken together, these evolutionary young, poorly conserved, and genomically atypical sequences point at a class of snoRNA-like transcripts with predominantly regulatory functions in the fruit fly genome. It is quite plausible that many, or even most, of the novel snoRNAs do not have canonical functions in rRNA or snRNA processing. Instead, effects on splicing or a role as precursor of microRNA-like small RNAs might well be their primary biological function. This view is supported by the disparate developmental expression patterns in Figure 3. Out of eight assayed snoRNAs, three are strongly upregulated from embryo to adult, the three members of *Uhg46E3* are significantly downregulated, and only two snoRNAs show a nearly constant expression pattern.

Finally, the findings reported here indicate that despite the plethora of available NGS data we have by no means a complete or comprehensive overview of the repertoires of structured regulatory RNAs—even in well-studied model organisms. Several effects may have conspired to hide the transcripts reported here: low sequence conservation, low or moderate expression levels, and rapid processing might have removed the loci from NGS analysis pipelines. At the same time a failure to exhibit characteristic features of Dicer-cleavage may have precluded the recoding of potential small processing products—the latter are nearly ubiquitous for snoRNAs [37,38]. The RNA deriving from coding sequences, of course, cannot be detected as independent entities by any of the usual NGS analysis work flows. We suspect that a large number of small and medium size RNAs with potentially important biological function still await discovery.

## Supplementary Materials

Supplementary materials can be accessed at: http://www.mdpi.com/2311-553X/1/2/139/s1.
