# Supplementary material for: Unusual Novel SnoRNA-Like RNAs in Drosophila melanogaster
Source: Noncoding RNA. 2015 Jul 13;1(2):139–50. doi: 10.3390/ncrna1020139 (PMC5932544; doi:10.3390/ncrna1020139)
Supplement: Supplementary file 1 [file ncrna-01-00139-s001.pdf]

Unusual Novel SnoRNA-Like RNAs in *Drosophila melanogaster*

Alberto Agrisani, Hakim Tafer, Peter F. Stadler and Maria Furia

Supplemental Material

Supplemental Table 1. Target predictions for snoRNA in ribosomal and spliceosomal RNAs. The interactions are shown with the snoRNA guide sequence in 3'→ 5' orientation on top and the target RNA sequence in 5'→3' orientation on the bottom. The predicted methylation site is indicated by a ^-character.

<table>
<tr><th>snoRNA</th><th>type</th><th>target</th><th>position</th><th>base pairing</th></tr>
<tr><td>*snoRNA:Me28S-A2629*</td><td>C/D</td><td>28S rRNA</td><td>A2629</td><td>AGAUUGCUCGAA
 :||||||| ||
AUUAACGAGAUU
    ^</td></tr>
<tr><td>*snoRNA:Me28S-C2789*</td><td>C/D</td><td>28S rRNA</td><td>C2789</td><td>AGGAGCCAACUU
 |||||||| :|
ACCUCGGUUUGG
    ^</td></tr>
<tr><td>*snoRNA:Me18S-G1506*</td><td>C/D</td><td>18S rRNA</td><td>G1506</td><td>GACACAAAGUUA
:||||||| |||
UUGUGUUUGAAU
    ^</td></tr>
<tr><td>*scaRNA:MeU1:95C-A24*</td><td>C/D</td><td>U1 snRNA</td><td>A24</td><td>CAAUUGGUAUAAG
|||||||:|: ||
GUUAACCGUGAUC
    ^</td></tr>
<tr><td>*scaRNA:MeU4:25F-C137*</td><td>C/D</td><td>U4 snRNA</td><td>C137</td><td>CGGGGUUUCCGUUAC
|||||||: | : ||
GCCCCAAGUGGGCUG
    ^</td></tr>
<tr><td>*snoRNA:Me28S-C993*</td><td>C/D</td><td>28S rRNA</td><td>C993</td><td>ACUUGAUCUUAACGAG
||||||| | ||| ||
UGAACUAUACUUGAUC
    ^</td></tr>
<tr><td>Box D'</td><td></td><td>18S rRNA</td><td>C1686</td><td>UAGAGGAUCUAGU
||:||||||| |:
AUUUCCUAGACCG
    ^</td></tr>
</table>

scaRNA:MeU1:95C-A24 is predicted to recognize all Drosophila U1 snRNA isoforms at a sequence conserved in all of them.

Supplemental Table 2. Summary of the best putative targets for the orphan Box C/D snoRNAs. The binding energy in the ΔG column is given in kcal/mol.

Name	ΔG	Gene	Function
snoRNA:Or-CD13	-30.20	dlg1 (intron)	protein-binding
snoRNA:Or-CD14	-24.80	EC 3.1.1.3 (exon)	lipid catabolic process
snoRNA:Or-CD15	-31.50	Hel89B (intron)	immune response
snoRNA:Or-CD16	-34.40	cadN (exon)	axon extension
snoRNA:Or-CD17	-39.20	CG-34380 (intron)	signal transduction

Supplemental Figure 1. UCSC genome browser maps of the new snoRNAs and snoRNA-like RNAs reported in this contributions (thick black arrows). Gene annotation and conservation tracks are shown.

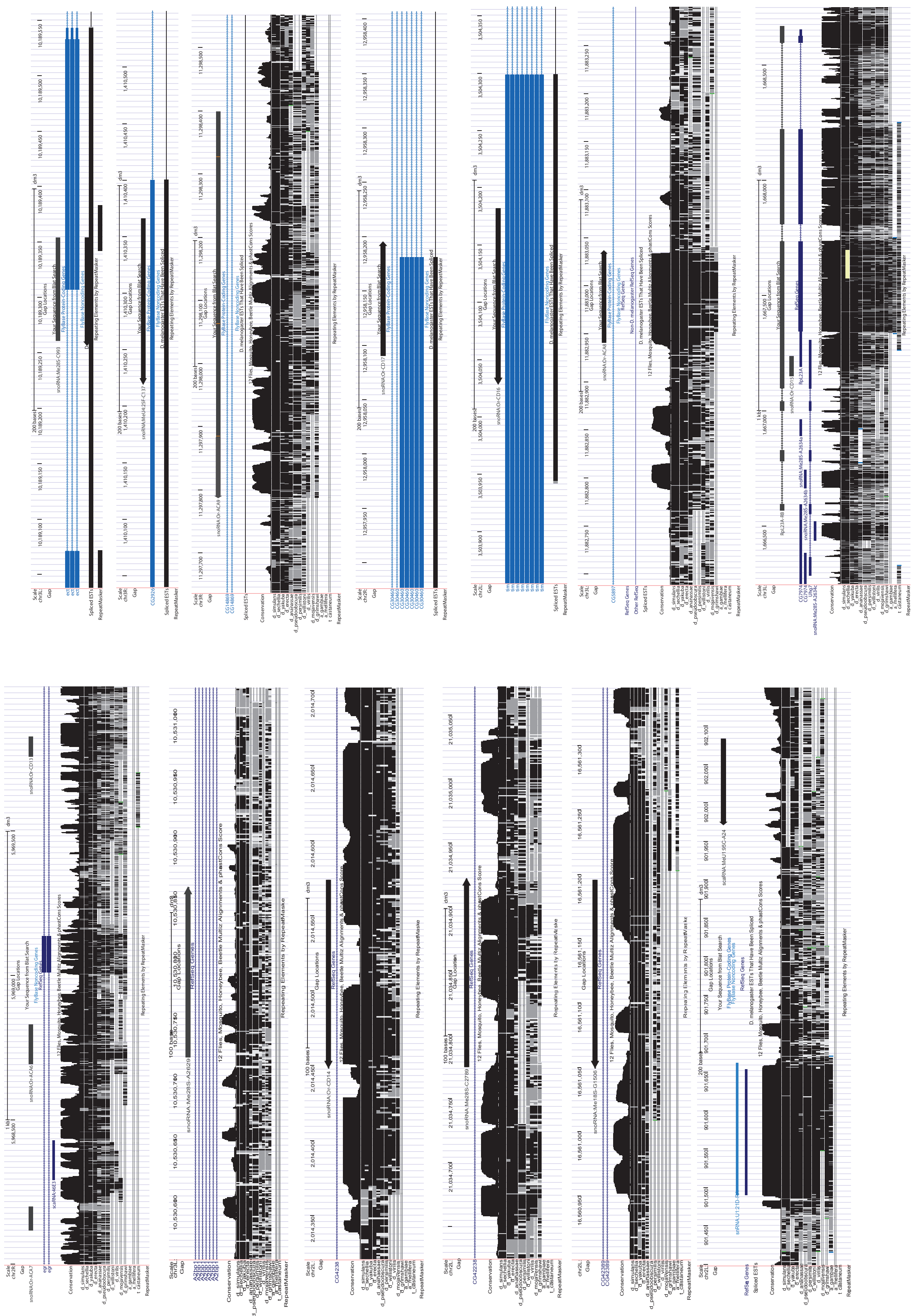

Supplemental Figure 2. Northern blots and RT-PCR of novel snoRNAs and snoRNA-like RNAs.Upper panel expression of snoRNAs derived from HG exons and/or characterized by uncommon length. Lower panel, Northern blot and RT-PCR expression of snoRNAs characterized by common length. In Northern blot Low range RNA Molecular Weight Markers (Fermentas), in RT-PCR DNA Molecular Weight Marker XIII (Roche) were used. Template for PCR: (g) gDNA, (+) RT+, (-) RT-; 7SL-RNA was used as positive control.

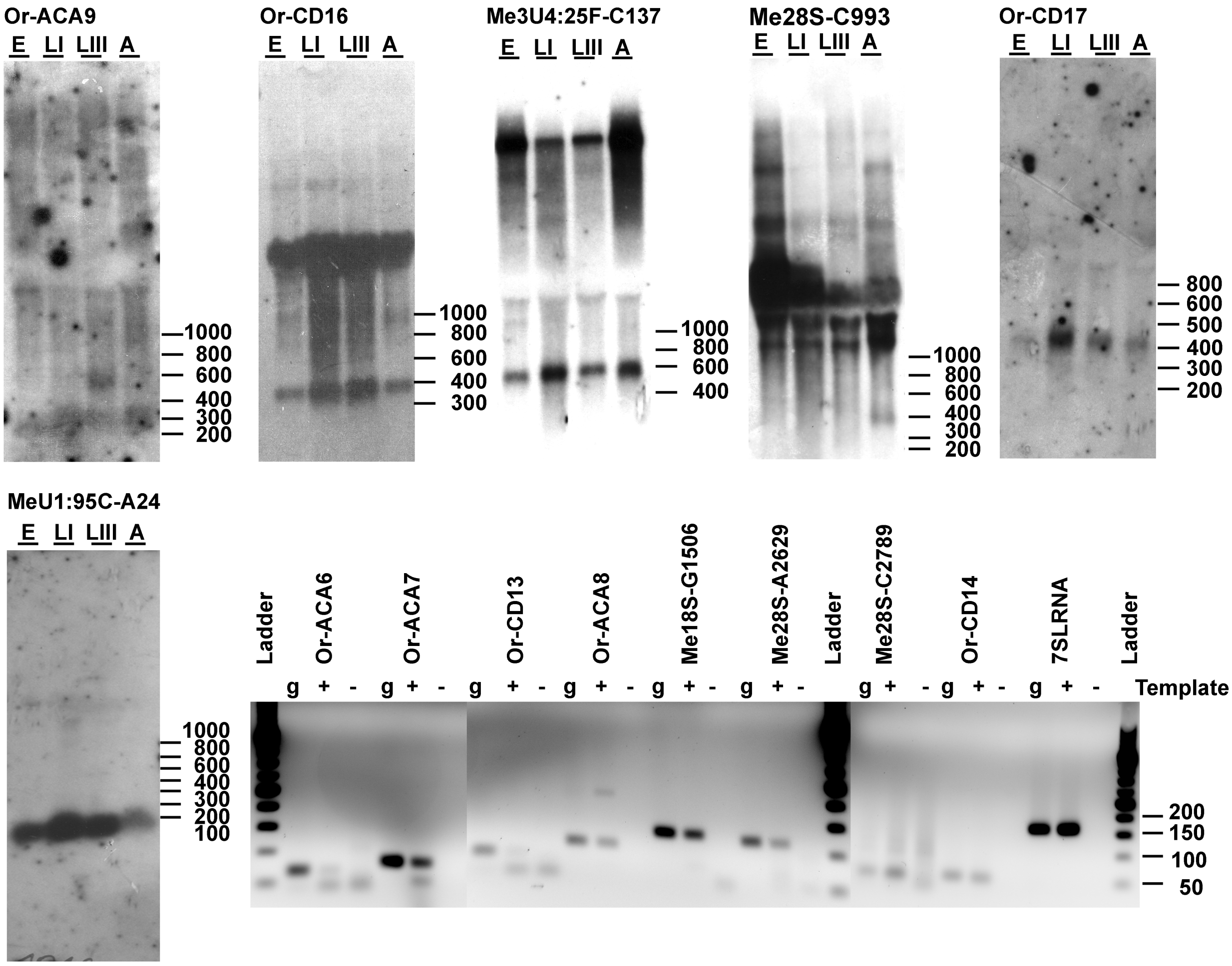

Supplemental Figure 3. Graphical representation of the analysis workflow leading to the identification of the 15 new snoRNAs.

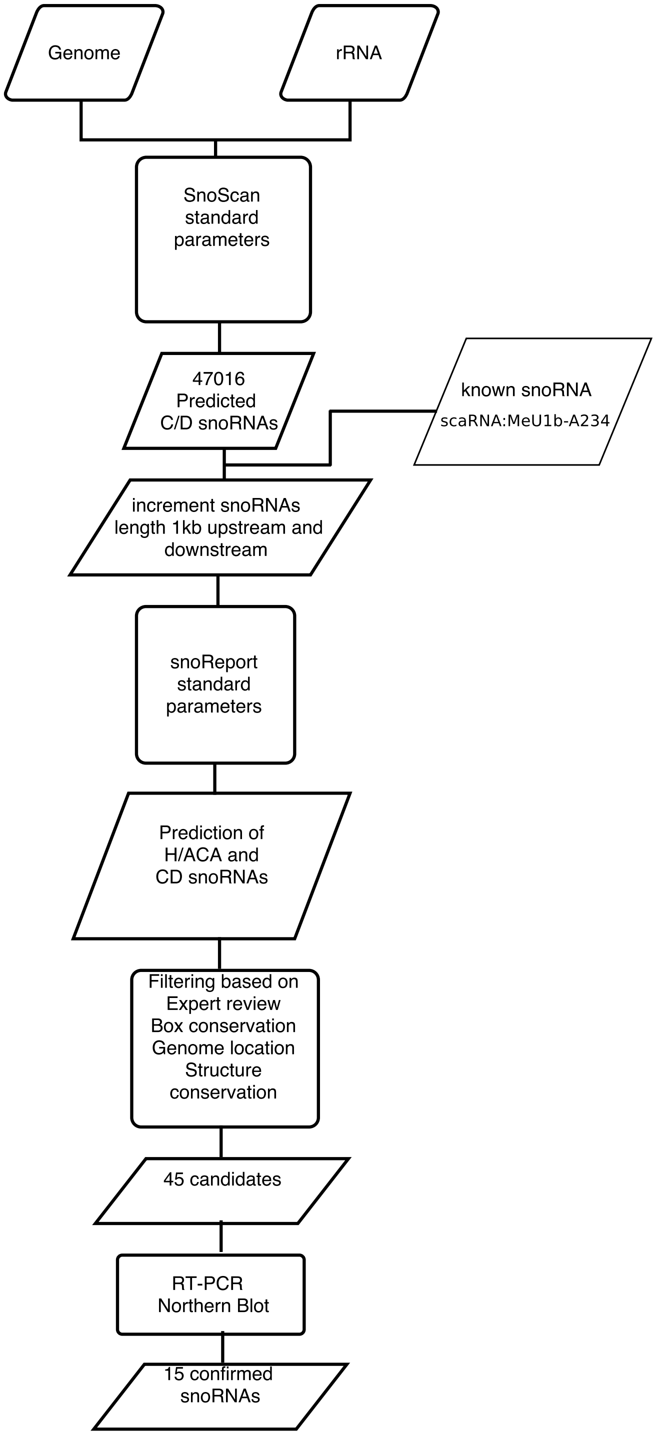

Supplemental Data File 1. Sequences of the four snoRNA-like RNAs not deposited in GenBank as identified by `SnoScan` and `SnoReport`. C/C' boxes are shown in italics, D/D' boxes in boldface.

> *snoRNA:Or-CD16*
ATGCCAAGG*ATGATG*CGGTCCTGGAGTCGGTCATCCGGATACTCGTCAAT**CTGA**CGGT
GCCGGTGGAGTGCCTCTTCTCCGTGGAC*GTGATGTA*CCGCACGGATGTGGGTCGCCACA
CCATCTTCGAG**CTGA**ACAAGCTGCTGTACACCAG
> *scaRNA:MeU4:25F-C137*
TAGATG*ATGATGA*TGAGGATACGGATATACCATTGCCTTTGGGGC**CTGA**GCCCGAACCC
GTTCCCGAAAAAGTCCCGAAAAGTAGTG*ATGATGA*CCTCTACGAGCCAGAGAATCCAAC
CGAAGAAC**CTGA**AGAACCCGAAATG
> *snoRNA:Me28S-C993* box D Me18S-C1686 box D'
CGACG*ATGATGA*GCCTGATCTAGGAGAT**CTGA**TCGATGACG*ATGATGG*TAGGTTTAG
AGCAATTCTAGTTCA**CTGA**ATGCACCCAGGCCGCAC
> *snoRNA:Or-CD17*
TGACGCACTAGGGTTG*CTGATGA*TTGGTGCTTGGAGACCAGACTACCGATTCCCTGCT
CATCCATTTCCACATTGCAAATGGGCGTGCCGC**CTGA**TGTGGAAC
